# Convergent Gut Microbiome Remodeling Across Ischemic Stroke, Myocardial Infarction, and Longevity Reveals a Shared Ecological Signature of Aging and Disease

**DOI:** 10.3390/ijms27157020

**Published:** 2026-08-05

**Authors:** Chuisheng Zeng, Jianfang Chen, Xuetong Yong, Yongfang Xie

**Affiliations:** 1 Chongqing Key Laboratory of Big Data for Bio Intelligence, Chongqing University of Posts and Telecommunications, Chongqing 400065, China; zengcs@cqupt.edu.cn (C.Z.); s250701002@stu.cqupt.edu.cn (J.C.); yongxuetong@outlook.com (X.Y.); 2 School of Life Health Information Science and Engineering, Chongqing University of Posts and Telecommunications, Chongqing 400065, China

**Keywords:** gut microbiota, ischemic stroke, myocardial infarction, aging, longevity-associated microbiome, age stratification, 16S rRNA sequencing

## Abstract

Gut microbiota dysbiosis has been associated with ischemic stroke (IS), myocardial infarction (MI), and aging, but whether these contexts share reproducible microbial features remains unclear. We conducted an exploratory and hypothesis-generating descriptive study of genus-level microbiota patterns across an internal IS cohort and publicly available external IS, MI, and age-stratified or longevity-associated datasets. Analyses were performed within predefined age strata and interpreted cautiously because of the small internal cohort, cross-cohort heterogeneity, and the absence of direct metabolite, intestinal barrier, inflammatory, or microbial activity measurements. No taxon in the internal cohort remained statistically significant after false-discovery-rate correction; therefore, all taxonomic observations were treated as descriptive. Candidate overlapping features included repeated detection of *Escherichia-Shigella* and *Klebsiella* and non-uniform patterns among genera previously associated with short-chain fatty acid metabolism, including *Faecalibacterium*, *Blautia*, and *Roseburia*. *Lachnoclostridium* and *Bacteroides* showed opposite abundance gradients in selected cross-dataset comparisons. These observations suggest possible ecological overlap across ischemic disease and age-associated microbiome contexts, but they do not establish causality, disease-specific biomarkers, or shared microbial function. The mechanistic models discussed in this manuscript are literature-informed hypotheses based on exploratory compositional data and require future validation in larger, harmonized longitudinal cohorts using metagenomic, metabolomic, clinical, and experimental measurements.

## 1. Introduction

Ischemic stroke (IS) remains a leading cause of mortality and long-term disability worldwide. Although advances in reperfusion strategies, including endovascular therapy for eligible patients with acute large-vessel occlusion, have improved clinical outcomes, IS is still associated with systemic inflammatory, metabolic, and immune alterations beyond cerebral injury [1]. Increasing evidence suggests that gut microbiota dysbiosis is associated with IS, myocardial infarction (MI), and age-associated physiological changes. However, microbiome profiles often vary substantially across cohorts, and differences in sequencing platforms, population characteristics, and analytical workflows may obscure reproducible disease-associated signals. This lack of reproducibility remains a major methodological challenge in microbiome research [2].

To address this issue, we first examined genus-level taxonomic patterns across an internal IS cohort and a publicly available external IS cohort and then compared the commonly detected genera with those observed in MI and longevity- and age-associated gut microbiome populations [3]. Because microbial differences across age strata may overlap with those observed between disease groups, cross-disease comparisons were performed within predefined age-matched strata to reduce the influence of age-related ecological variation [4].

Genera commonly associated with short-chain fatty acid (SCFA) metabolism were interpreted cautiously in this study. SCFA biosynthetic capacity can vary among species and strains within the same genus and is influenced by dietary substrates, microbial cross-feeding, intestinal conditions, and baseline community composition [5,6]. Therefore, genus-level abundance patterns alone cannot be considered direct evidence of altered intestinal SCFA production [7].

Based on these considerations, we hypothesized that selected genus-level microbial patterns, including SCFA-associated genera and putative opportunistic taxa, may show partial overlap across IS, MI, and longevity- and age-associated gut microbiome populations within predefined age strata [8]. The 60–80-year range was selected as the principal overlapping age range across the included cohorts to improve age comparability, rather than as a presumed biological threshold. Because fecal or circulating SCFA concentrations and microbial metabolic activity were not directly measured, all SCFA-related and functional interpretations were regarded as hypothesis-generating compositional observations rather than direct evidence of altered microbial metabolic activity [9].

## 2. Results

### 2.1. Gut Microbial Diversity in Ischemic Stroke Subgroups

To assess the overall ecological structure of the gut microbiota during the stroke and after treatment, this study first conducted a comparative analysis of three subgroups within the internal IS cohort: the patient group (*n* = 33), the treatment group (*n* = 19), and the healthy control group (*n* = 21). The genus-level relative-abundance matrix for the internal IS cohort was used for this analysis (Table S5).

**Figure 1:**
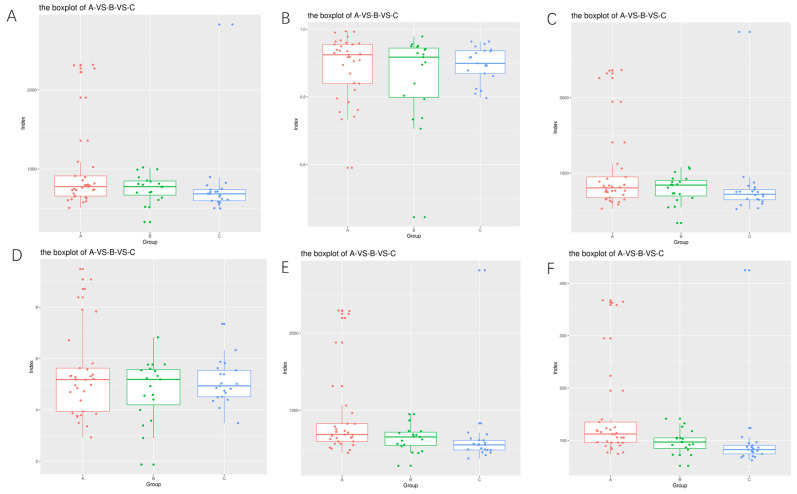
Alpha diversity analysis of gut microbiota in the internal ischemic stroke (IS) cohort. Boxplots showing six alpha-diversity indices among the patient (A), treatment (B), and healthy control (C) groups: (**A**) Sobs, (**B**) Pielou’s evenness, (**C**) Chao1, (**D**) Shannon, (**E**) ACE, and (**F**) Simpson. The treatment group exhibited higher richness-related indices than the patient group, whereas evenness-related indices showed no marked differences among groups.

**Figure 2:**
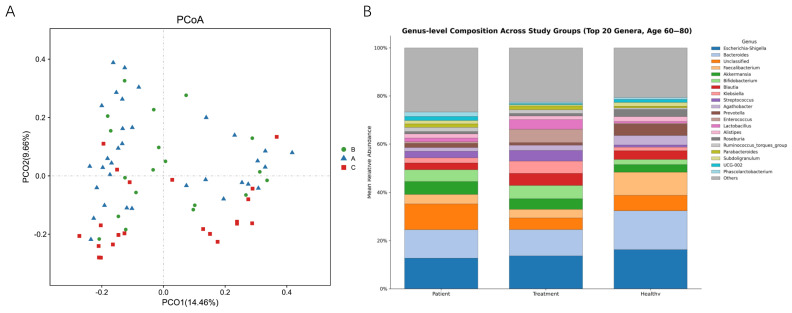
Microbial community structure and taxonomic composition in the internal ischemic stroke (IS) cohort. (**A**) Principal coordinates analysis (PCoA) based on Bray–Curtis distances showing microbial community differences among the patient, treatment, and healthy control groups. (**B**) Mean relative abundances of the top 20 bacterial genera in the three groups. The treatment group exhibited partial restoration of microbiota composition compared with the patient group.

Alpha diversity analysis revealed differences in richness indices among groups, whereas evenness indices showed no significant differences (Figure 1). The treatment group exhibited higher richness than the patient group (PD, *p* = 0.019), suggesting that post-treatment microbial changes were primarily characterized by a selective increase in richness. PCoA showed that samples from the three groups did not form distinct clusters, with the first two axes explaining 22.54% of the variance (Figure 2A). The genus-level relative abundances of the top 20 bacterial genera across the three groups are shown (Figure 2B) Although PERMANOVA indicated significant differences between groups (*R*^2^ = 0.060, *p* = 0.001), group membership explained only 6.0% of the variance, and the *R*^2^ values for all pairwise comparisons were below 0.07 [10]. ANOSIM detected significant differences only between the healthy control group and the patient group (*R* = 0.125, *p* = 0.002). PERMANOVA results and related community-structure statistics are summarized (Table S9). This inconsistency may reflect differences in the sensitivity of the two methods to within-group dispersion [11].

Taken together, the alpha- and beta-diversity results indicated modest differences in microbial community structure among the IS subgroups [12]. Although partial separation was observed, the overall overlap in community composition and the small PERMANOVA effect size suggest that the detected differences should be interpreted as exploratory rather than evidence of extensive community restructuring. Specifically, differences in richness indices coexisted with only small differences in community composition. These findings suggest that stroke-associated dysbiosis, under the current sample size, is mainly characterized by selective changes in specific taxonomic groups [13] rather than a large-scale reorganization of the microbial community. This observation provides a basis for subsequent analyses focusing on reproducible microbial signals at the genus level and cross-disease comparisons of predicted functional modules [14].

### 2.2. Cross-Cohort Comparison of Stroke-Associated Microbial Patterns

To establish an internal reference for stroke-associated gut microbiota alterations, this study first compared the baseline IS group, matched follow-up group, and healthy control group. Alpha-diversity analysis showed significant differences in richness indices, including Sobs and Chao1, among the three groups, with higher richness observed in the follow-up group than in the baseline IS group. In contrast, Shannon, Simpson, and Pielou indices did not differ significantly among groups (all *p* > 0.05), suggesting that the observed differences were mainly related to richness rather than community evenness [15]. The internal IS genus-level abundance matrix supported these comparisons (Table S5).

PCoA analysis based on Bray–Curtis distances showed partial overlap among the three groups. PERMANOVA detected a statistically significant difference in community composition (*R*^2^ = 0.060, *p* = 0.001), although the effect size was modest, indicating that group membership explained only a small proportion of the overall variation. PERMDISP showed no significant difference in within-group dispersion (*p* > 0.05), suggesting that the PERMANOVA result was unlikely to be driven by unequal dispersion among groups. ANOSIM identified a significant difference only between healthy controls and baseline IS patients (*R* = 0.125, *p* = 0.002). Together, these findings indicate modest community-level differences among groups and should be interpreted as exploratory (Table S9).

To examine whether similar genus-level patterns could be observed in an independent dataset, 145 participants aged 60–80 years from a publicly available external IS cohort were included for descriptive comparison [16]. Dataset characteristics and sample-level accession mapping are summarized (Tables S1 and S2). Alpha-diversity, PCoA, genus-level composition, and the corresponding genus-level abundance matrix for the external IS cohort are shown (Figures S1, S3 and S5; Table S6). PCoA based on Bray–Curtis distances also showed substantial overlap among samples in the external cohort, and PERMDISP did not detect significant heterogeneity in within-group dispersion (Bray–Curtis: F = 1.337, *p* = 0.131; Jaccard: F = 1.175, *p* = 0.092). The corresponding PERMANOVA results are summarized (Table S9).

Taxonomic profiles were generated independently for the internal and external IS cohorts and compared at the genus level. Fifteen genera exceeding a mean relative abundance of 0.1% were descriptively detected in both the internal IS cohort (n = 33, aged 60–80 years) and the external IS cohort (n = 145, aged 60–80 years), including *Bacteroides*, *Escherichia-Shigella*, and *Blautia*. These overlapping genera were regarded as candidate commonly detected genera across stroke cohorts, rather than validated disease-specific microbial signatures [17].

From an ecological perspective, facultative anaerobic opportunistic pathogens (*Escherichia-Shigella* and *Klebsiella*) were consistently detected in both independent stroke cohorts, whereas genera associated with SCFA metabolism (*Faecalibacterium* and *Blautia*) were stably present at relatively low abundances [18]. This ecological pattern, characterized by the persistent presence of opportunistic pathogens and a relative deficiency of SCFA-producing commensals, was observed in both datasets. These findings suggest that the cross-cohort consistency of stroke-associated dysbiosis may be better reflected in directional shifts at the functional niche level rather than strict replication of the abundance of individual genera [19].

### 2.3. Exploratory Age-Stratified Cross-Cohort Taxonomic Overlap Between Ischemic Stroke and Myocardial Infarction

Building on the reproducible genera identified across stroke cohorts, this study further evaluated whether shared microbial characteristics between ischemic stroke and myocardial infarction differed across age strata. Analyses were conducted separately within two age-matched groups: 40–60 years, represented by the external IS cohort, and 60–80 years, represented by the combined internal and external IS cohorts. Because only seven genera in the MI cohort exceeded a mean relative abundance of 0.1% under this descriptive filtering criterion, subsequent cross-disease comparisons were limited to this subset. The MI cohort alpha-diversity, PCoA, genus-level composition, and genus-level abundance matrix are shown (Figures S2, S4 and S6 and Table S7).

In the 40–60-year age group, 14 genera exceeded the 0.1% mean relative abundance threshold in the external IS cohort under the same descriptive filtering criterion, whereas the MI cohort contained seven. All seven MI genera were also identified in the external IS cohort, including *Bacteroides* and *Dorea*. In contrast, the external IS cohort contained several low-abundance genera that did not reach the same detection threshold in the MI cohort, including *Prevotella* and *Eubacterium*. These findings indicate substantial genus-level overlap between the two disease cohorts under the predefined abundance threshold [20,21]. The abundance matrices used for this comparison are shown (Tables S6 and S7). *Lachnoclostridium* was more abundant in MI than in stroke, suggesting that disease-related differences in specific genera remained despite the shared taxonomic framework [22].

Similar analyses were performed in the 60–80-year age group. The internal and external IS cohorts shared 15 reproducibly detected genera. After comparison with the MI cohort, six genera were retained across all three disease cohorts, including *Bacteroides* and *Escherichia-Shigella*. Compared with the 40–60-year group, *Dorea* in the 60–80-year MI cohort fell below the detection threshold and was therefore not included in the shared cross-disease set, suggesting possible age-stratum-related differences [23]. The abundance matrices used for this comparison are shown (Tables S5–S7).

Within the predefined 60–80-year stratum, the retained six-genera overlap should be interpreted as a descriptive cross-cohort observation rather than evidence of an age-driven microbial convergence process. Because the contributing cohorts differed in disease characteristics, recruitment settings, geographic background, sequencing procedures, and the availability of medication, diet, and lifestyle information, the effects of age could not be fully separated from cohort-related and clinical effects.

**Figure 3:**
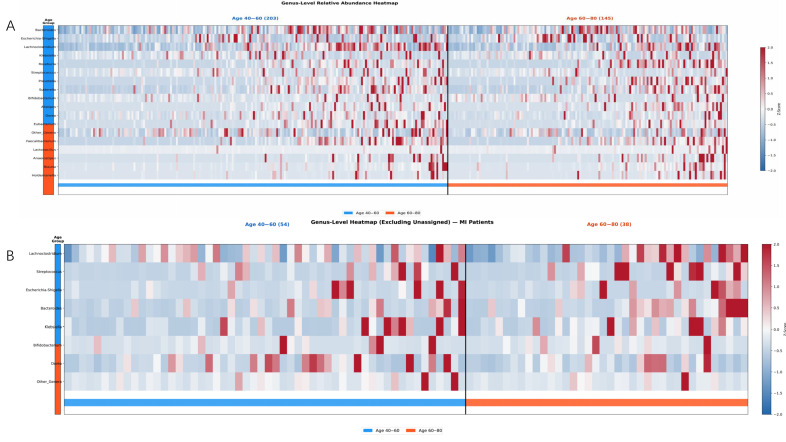
Age-stratified genus-level heatmaps of gut microbiota in ischemic stroke and myocardial infarction cohorts. (**A**) Genus-level relative abundance heatmap of the external ischemic stroke (IS) cohort, stratified into the 40–60 and 60–80 age groups. (**B**) Genus-level relative abundance heatmap of the myocardial infarction (MI) cohort, stratified into the 40–60 and 60–80 age groups. Colors represent standardized relative abundance values (Z-scores), with red indicating relatively higher abundance and blue indicating relatively lower abundance.

At the genus level, the shared microbial community structure between stroke and myocardial infarction appears relatively stable. Six genera, including *Bacteroides* and *Escherichia-Shigella*, were consistently detected across both age groups [24], whereas *Dorea* showed age-dependent exclusion. However, abundance patterns within this shared framework were not uniform. *Lachnoclostridium* remained consistently abundant in MI across age groups, whereas *Bifidobacterium* varied considerably between the internal and external IS cohorts. These results suggest the presence of a stable ecological framework shared across ischemic diseases, rather than representing all microbial differences between diseases [25]. The corresponding age-stratified genus-level abundance patterns in the external IS and MI cohorts are presented (Figure 3).

### 2.4. Common Gut Microbial Alterations Across Ischemic Disease and Age-Stratified Gut Microbiome Datasets

Building on the shared genera identified in stroke and MI, this study further incorporated longevity- and age-associated gut microbiome datasets (*n* = 2650) to evaluate whether the identified microbial framework represented conserved ecological patterns beyond specific disease contexts. Genus-level annotation coverage was higher in the longevity- and age-associated datasets than in the MI cohort. The genus-level composition and abundance matrix for these age-stratified datasets are shown (Figure S7; Table S8). PCoA analysis revealed partial convergence in microbial community structure across the analyzed dataset categories, with PERMANOVA results summarized (Table S9). Subsequent analyses were conducted separately within the 40–60 and 60–80 age groups using the same age-matching strategy.

In the 40–60 age group, intersection analysis among the external IS cohort, MI cohort, and longevity- and age-associated gut microbiome datasets identified seven shared genera. All seven genera identified between the external IS and MI cohorts were consistently detected in the longevity- and age-associated datasets. Notably, *Bacteroides* maintained a high abundance gradient across these groups (external IS > MI > longevity-associated populations), whereas *Lachnoclostridium* showed marked differences in abundance among diseases. *Escherichia-Shigella* and *Klebsiella* were detected in all three diseases, although their abundance levels varied by disease.

**Figure 4:**
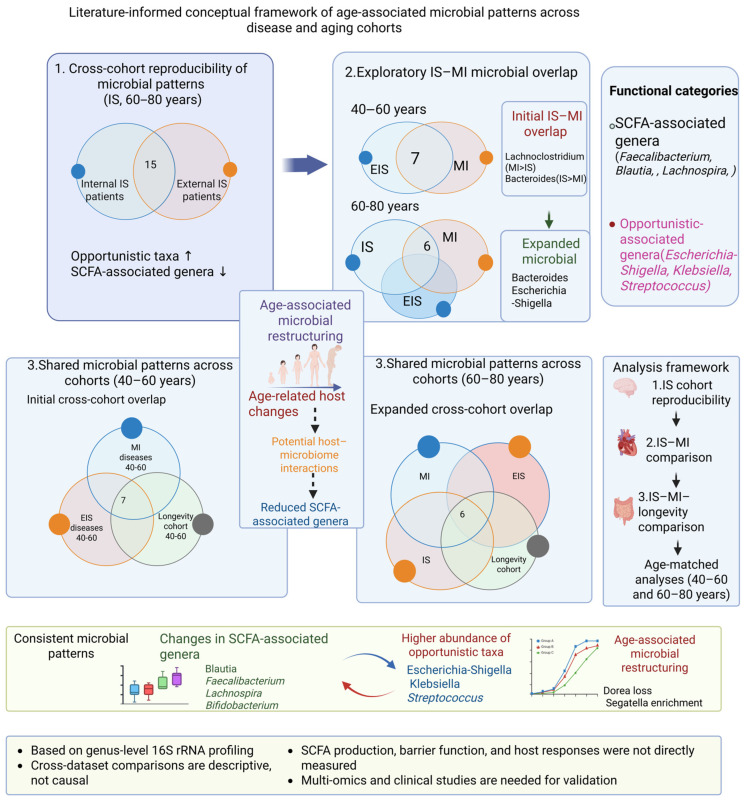
Literature-informed conceptual framework of shared gut microbial patterns across ischemic stroke, myocardial infarction, and age-associated populations. The framework summarizes exploratory genus-level overlaps, including opportunistic-associated taxa, SCFA-associated genera, and Lachnoclostridium abundance patterns across cohorts. The proposed interpretations are literature-informed and hypothesis-generating.

In the 60–80 age group, a four-level progressive intersection analysis incorporating the internal IS cohort, external IS cohort, MI cohort, and longevity- and age-associated gut microbiome datasets revealed a similar pattern. Of the 15 genera shared between the internal IS and external IS cohorts, six were retained after intersection with the MI cohort. After further incorporation of the longevity- and age-associated gut microbiome datasets, these retained genera were evaluated to determine whether the observed microbial patterns extended beyond disease-specific contexts; all six genera remained detectable under the predefined abundance criterion, forming the final retained cross-dataset genus set. This progressive overlap is visualized with the supporting genus-level matrices (Figure S8; Tables S5–S8). In terms of abundance patterns, *Bacteroides* showed a higher relative abundance in longevity- and age-associated gut microbiome datasets compared with MI, whereas *Lachnoclostridium* exhibited the opposite trend and was consistently detected across analytical comparisons, showing a stable abundance gradient (MI > stroke > longevity-associated populations). The *Lachnoclostridium* abundance gradient is shown (Figure S9). Across both age groups, six common genera, including *Bacteroides* and *Escherichia-Shigella*, were consistently retained, representing a descriptive microbial pattern shared across ischemic stroke, myocardial infarction, and age-associated gut microbiome populations. These cross-dataset taxonomic patterns and their literature-informed biological context are summarized in the conceptual framework (Figure 4).

### 2.5. Treatment-Related Changes in Microbial Features

To evaluate longitudinal changes in the microbial community, baseline samples from patients, matched follow-up samples from the same 19 patients, and healthy controls were compared. Distance-based beta-diversity analysis indicated persistent differences in community structure between follow-up samples and healthy controls, whereas follow-up samples remained more similar to their corresponding baseline samples [26]. These findings suggest that, over the follow-up period, the overall gut microbial community composition remained distinct from that of healthy controls. Because the analysis was based on a limited number of matched longitudinal samples and detailed treatment information was not consistently available, these observations should be interpreted as exploratory and should not be regarded as evidence of microbiome restoration or treatment efficacy [27].

At the genus level, several shared genera showed partial shifts toward control-like abundance levels. The relative abundance of *Escherichia-Shigella* was lower in the follow-up group than in the baseline patient group and approached the level observed in healthy controls. In contrast, *Faecalibacterium* and *Roseburia* increased in relative abundance, although their levels remained below those of healthy controls. The follow-up group also showed higher richness indices, including Sobs and Chao1, than the baseline patient group, with some values exceeding those observed in healthy controls. These findings indicate post-treatment differences in alpha diversity and selected genera, as supported by the internal cohort abundance matrix (Table S5).

Despite the increase in alpha diversity and partial genus-level shifts, community structure did not fully converge toward that of healthy controls [28]. Together with the beta-diversity results, these observations suggest that changes in selected shared genera and richness indices may occur without complete normalization of overall community structure, as reflected by the PERMANOVA results (Table S9).

### 2.6. Age-Gradient Structure of the Gut Inflammatory Microbiota

**Figure 5:**
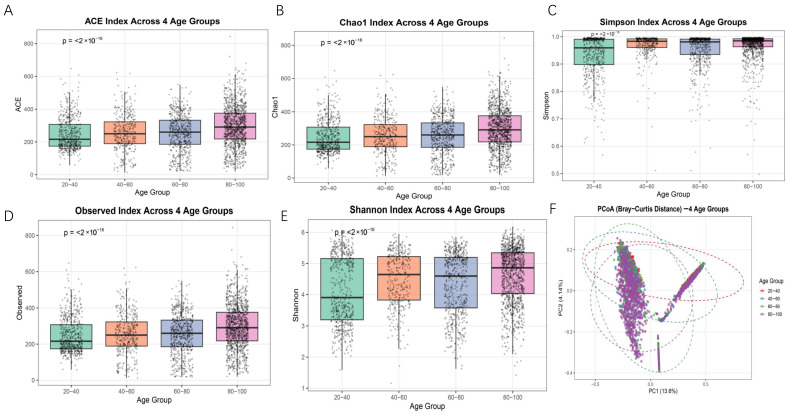
Gut microbiota diversity and community structure across predefined age strata. (**A**–**E**) Boxplots of ACE, Chao1, Simpson, Observed species, and Shannon indices across four age groups (20–40, 40–60, 60–80, and 80–100 years). (**F**) PCoA based on Bray–Curtis distances showing age-associated variation in microbial community composition. All α-diversity indices differed significantly among age groups (*p* < 2 × 10^−16^).

By incorporating individuals across the 20–40, 40–60, 60–80, and 80–100 age strata from longevity- and age-associated gut microbiome datasets, this study established an age-gradient framework to investigate age-related microbial remodeling. The primary purpose was to provide an independent ecological context for exploratory cross-cohort comparisons and to facilitate comparisons within the predefined 60–80-year age stratum. Alpha-diversity analysis revealed significant differences in both the Shannon and Simpson indices across the four age groups, indicating that gut microbiota diversity varied with age (Figure 5A–E) [29]. The source abundance matrix and genus-level composition for these age-stratified datasets are shown (Table S8; Figure S7). As age increased, median community richness showed a gradual stratification pattern [30], whereas inter-individual variability increased markedly in the 80–100 age group, suggesting greater heterogeneity in microbiota composition during advanced aging [31].

PCoA analysis revealed differences in community composition among the four predefined age strata (Figure 5F). The distance in community structure between the 60–80 and 20–40 age groups was greater than that observed between adjacent age groups, indicating that substantial age-related microbial remodeling had occurred by this stage [32]. PERMANOVA results for the age-stratified datasets are summarized (Table S9). At the taxonomic level, *Escherichia-Shigella* increased progressively with age (1.8% in the 20–40 age group to 11.4% in the 80–100 age group). In contrast, *Prevotella/Segatella* decreased with age (14.99% to 4.34%), consistent with the reduced abundance of SCFA-associated taxa observed across diseases [33]. Age-associated differential genera are summarized (Table S10).

**Figure 6:**
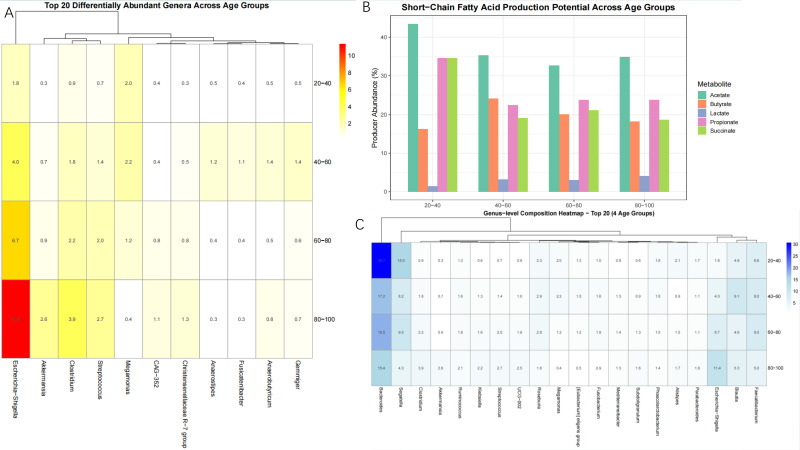
Age-stratified differences in gut microbiota composition and predicted metabolic functions. (**A**) Differential genus abundance heatmap across age groups. (**B**) Predicted SCFA production potential. (**C**) Heatmap of KEGG pathway abundances. Opportunistic pathogens (*Escherichia-Shigella* and *Klebsiella*) increased with age, whereas SCFA-associated genera (*Faecalibacterium* and *Bifidobacterium*) remained relatively low. Functional prediction suggested lower predicted acetate and butyrate production potential in the older age strata, accompanied by decreased carbohydrate and energy metabolism pathways and increased lactate- and succinate-associated functions.

PICRUSt2 functional prediction suggested differences in the predicted functional profiles of the gut microbiota across the predefined age strata [34]. The corresponding age-stratified taxonomic differences and predicted SCFA- and KEGG-related functional profiles are shown (Figure 6A–C). Pathways involved in carbohydrate degradation and polysaccharide utilization declined significantly with age (FDR = 2.80 × 10^−45^) [35], and butyrate production further decreased in the 80–100 age group (FDR = 4.05 × 10^−37^), consistent with the lower relative abundance of SCFA-producing genera, such as *Blautia*, observed in the older age stratum. In contrast, spore formation (FDR = 2.16 × 10^−61^) and lactic acid fermentation pathways were significantly enriched in older individuals [36], consistent with the increased abundance of *Streptococcus*. The age-associated functional pathway summaries are shown (Table S11).

Overall, the gut microbiota in older individuals showed a functional shift from carbohydrate metabolism toward pathways associated with environmental adaptation and survival [37,38]. Combining the taxonomic and predicted functional results, individuals in the predefined 60–80-year age stratum exhibited distinct microbial community characteristics, providing independent ecological support for selecting this age range as a window for cross-system comparisons.

These findings characterize age-stratified compositional variation within longevity- and age-associated gut microbiome datasets and provide contextual support for, but do not independently validate, the proposed descriptive cross-cohort age-stratified pattern [39]. Moreover, PICRUSt2-predicted butyrate-related functional potential was broadly stable between 40 and 80 years and showed a more evident decrease in the 80–100-year group; therefore, it does not support a specific functional transition at 60–80 years [40].

## 3. Discussion

### 3.1. The Methodological Value of a Reproducibility-Focused Framework

Existing cross-cohort microbiome studies are often limited by cohort heterogeneity and poor reproducibility. To reduce these sources of bias, this study predefined a stepwise comparison strategy and criteria for identifying shared microbial features before analysis [41]. All external cohorts were reprocessed using a standardized bioinformatics workflow, and only signals reproducibly detected across datasets were included in subsequent comparisons [42], thereby reducing cohort-specific noise.

Using this approach, 15 genera were consistently detected in both the internal and external IS cohorts within the 60–80–year age group. Pro-inflammatory facultative anaerobes and genera associated with SCFA metabolism showed consistent enrichment and depletion patterns, respectively, across the two cohorts [43], whereas non-reproducible genera were excluded from subsequent analyses. This reproducibility-based filtering strategy may help reduce false-positive findings and improve the robustness of cross-cohort comparisons [44].

However, the resulting findings should still be regarded as descriptive ecological observations. Although most cross-disease overlaps did not reach statistical significance, the repeated identification of similar microbial patterns across different levels of analysis suggests that this framework may have methodological value. Future studies incorporating larger cohorts and higher-resolution sequencing approaches may further improve its ability to identify stable cross-disease microbiome signals.

### 3.2. Shared Microecological Signatures of Systemic Ischemic Diseases

Stroke and myocardial infarction (MI) exhibited substantial overlap in the identifiable portion of the gut microbiota. Specifically, all genera detected in the MI cohort were also detected in the stroke cohort. Seven genera were shared in the 40–60–year age group, and six of these remained in the 60–80–year age group, suggesting that this overlap is unlikely to be a chance finding driven by age alone [45]. However, a shared set of genera does not imply complete similarity in microbial community structure. For example, the abundance patterns of *Bacteroides* differed between the two diseases, indicating that microbiota alterations associated with ischemic diseases do not follow a single trajectory of dysbiosis [25], but instead result in distinct ecological configurations shaped by disease-specific pathophysiological environments under common inflammatory pressures. These observations suggest that the shared genera may reflect common ecological responses rather than identical microbial configurations.

From a functional perspective, the shared microbial alterations observed in these two ischemic conditions may involve remodeling of microbial networks associated with intestinal barrier homeostasis. In a large-scale multi-omics study (*n* = 548), Xu et al. (2025) reported stage-specific changes in the gut microbiome during CAD progression [46]. MI patients showed enrichment of pro-inflammatory taxa such as *Streptococcus* and elevated circulating levels of 3-hydroxybutyrate, whereas these microbial and metabolic characteristics returned to levels resembling stable CAD following clinical recovery [47]. These findings suggest that the gut microbiome and its metabolic output may respond dynamically to acute ischemic events. Zhou et al. (2018) further provided evidence for the translocation of gut-derived bacteria into the systemic circulation of STEMI patients, indicating that disruption of intestinal barrier integrity may contribute to systemic inflammation during ischemic cardiac events [48]. Taken together, the above evidence and the shared dysbiosis patterns observed in this study suggest that impaired gut barrier homeostasis and the resulting enteric inflammatory burden may represent an important pathological background underlying shared microbial alterations across ischemic diseases [49,50,51]. In ischemic stroke, whether gut-derived inflammatory signals contribute to the amplification of neuroinflammation through effects on blood–brain barrier homeostasis remains to be clarified.

The results of this study support the existence of a partially shared basis of gut microbiota alterations between stroke and myocardial infarction. The main features were characterized by differences in microbial community structure across predefined age strata and exploratory taxonomic overlap among the analyzed cohorts. However, substantial differences in the abundance profiles of shared genera between the two diseases indicate that microbiota reorganization associated with ischemic diseases does not represent complete microbiome convergence [12,25]. The clinical significance of this shared framework should be further evaluated in larger cohorts incorporating metagenomic analyses and longitudinal intervention studies [52].

### 3.3. Descriptive Cross-Disease Microbial Patterns and Their Potential Biological Context

#### 3.3.1. Convergent Expansion of Pro-Inflammatory Opportunistic Enterobacteriaceae

The hierarchical intersection analysis identified a retained set of potentially opportunistic taxa shared across ischemic stroke (IS), myocardial infarction (MI), and longevity- or age-associated gut microbiome populations. *Escherichia-Shigella* and *Klebsiella* emerged as repeatedly detected genera across the analyzed cohorts and age strata, forming a descriptive inflammation-associated taxonomic pattern linked to cross-dataset microbiome dysbiosis. Both genera belong to the Enterobacteriaceae family and are Gram-negative facultative anaerobes [53]. Their relative abundance generally increased with age across the analyzed cohorts, suggesting that this pattern may represent a recurring ecological feature across multiple inflammation-related contexts rather than a stroke-specific signature [54,55]. The retained overlapping genera and cross-dataset abundance patterns are shown (Figures S8 and S9).

The biological interpretation of this pattern is supported by, but not directly tested against, mechanisms proposed in previous studies. Lipopolysaccharides derived from *Escherichia-Shigella* and *Klebsiella* may enter the circulation when intestinal barrier integrity is impaired and may activate TLR4/NF-kB signaling, thereby contributing to systemic inflammatory responses [48,56,57]. Consistent with this hypothesis, Carnevale et al. detected *E. coli*-derived LPS in human carotid atherosclerotic plaques, with substantial overlap between LPS-positive regions and macrophage infiltration [58]. However, intestinal barrier integrity, circulating LPS, TLR4/NF-kB signaling, and inflammatory mediators were not measured in the present study. Therefore, these mechanisms should be interpreted as literature-based hypotheses requiring further experimental validation.

Independent cohort studies have also reported enrichment of related opportunistic taxa in inflammatory and cardiovascular disease settings. Haak et al. observed increased *Escherichia*/*Shigella* in stroke patients [59], Jie et al. reported enrichment of *Klebsiella* spp. in patients with atherosclerotic cardiovascular disease [60], and Zhou et al. provided evidence for translocation of gut bacteria into the systemic circulation of STEMI patients [48]. Together, these findings support the ecological relevance of opportunistic Enterobacteriaceae enrichment across inflammation-related conditions.

In summary, the repeated detection of *Escherichia-Shigella* and *Klebsiella* indicates a descriptive compositional pattern rather than a demonstrated functional mechanism [61]. These observations should not be interpreted as evidence of a conserved functional module across disease- and age-associated microbiome contexts, because functional readouts, including intestinal barrier measures, inflammatory signaling, and direct metabolite quantification, were not assessed in the present study [62]. Future studies combining strain-resolved metagenomics, direct metabolite measurement, and longitudinal sampling will be required to determine whether these compositional patterns correspond to consistent functional alterations across diseases.

#### 3.3.2. Taxonomic Patterns and Functional Heterogeneity of SCFA-Associated Genera

In the present study, *Faecalibacterium*, *Blautia*, and *Roseburia* were examined because these genera are frequently associated with short-chain fatty acid (SCFA) metabolism. Among genera passing the predefined abundance threshold, lower mean relative abundances of some of these genera were observed in selected within-dataset comparisons. However, these patterns were not consistent across all age strata and disease datasets, and not all three genera were consistently detected in the genus-level profiles of the MI dataset. Therefore, our findings do not support the co-depletion of a conserved SCFA-associated microbial module across disease- and age-stratified microbiome contexts. Instead, they indicate descriptive taxonomic changes involving selected SCFA-associated genera.

SCFA-associated genera should not be considered a functionally homogeneous group. SCFA biosynthetic capacity can vary substantially among species and strains within the same genus because microorganisms differ in carbohydrate-utilization pathways and SCFA-producing enzymes [63]. In addition, microbial SCFA production depends on dietary substrate availability and fiber structure, including solubility, molecular architecture, glycosidic linkages, and accessibility to microbial carbohydrate-active enzymes [64]. Consistent with this view, Fan et al. showed that soluble barley beta-glucans exerted donor-dependent effects on the fermentation of insoluble bran, suggesting that baseline microbiota composition can influence individual fermentation responses [65].

Although butyrate, acetate, and propionate have established roles in epithelial energy metabolism, luminal ecology, and mucosal immune regulation, genus-level relative abundance cannot determine SCFA production in vivo [9]. A lower relative abundance of *Faecalibacterium*, *Blautia*, or *Roseburia* therefore does not necessarily indicate reduced SCFA production, because functional output may be modified by species- and strain-level differences, substrate availability, intestinal transit, luminal conditions, microbial interactions, and host absorption. These findings should be interpreted as hypothesis-generating taxonomic observations rather than direct evidence of reduced SCFA biosynthesis, impaired epithelial function, or a shared SCFA-related mechanism across the examined cohorts. Future studies integrating shotgun metagenomics, metabolomic profiling, and direct SCFA quantification will be required to determine whether these taxonomic patterns correspond to functional alterations in microbial SCFA metabolism.

### 3.4. Exploratory Age-Stratified Patterns in Shared Microbial Genera

In age-stratified cross-cohort comparisons between the 40–60 and 60–80 groups, all six shared genera were consistently retained, suggesting that the observed overlap of selected microbial features across ischemic stroke (IS), myocardial infarction (MI), and longevity- and age-associated gut microbiome populations was maintained across different age strata. Importantly, this stability does not depend on the persistence of any single genus. Dorea showed clear age dependence, being retained only in the 40–60 age group and losing its shared status in older individuals. In contrast, Lachnoclostridium remained detectable across all age groups and disease comparisons, although its abundance exhibited a pronounced disease-dependent gradient [66,67]. Together, these observations suggest that some genus-level overlap may vary across predefined age strata while remaining detectable under the predefined abundance criterion. These descriptive observations may reflect shared ecological characteristics across disease cohorts; however, the present study was not designed to determine whether these similarities result from common inflammatory processes or other shared environmental and clinical factors [68,69].

Several biological mechanisms have been proposed in previous studies that may contribute to age-associated microbial remodeling. Previous studies have shown that the chronic low-grade inflammatory state associated with aging, commonly termed inflammaging, may increase intestinal oxidative stress and thereby enhance the ecological competitiveness of opportunistic facultative anaerobes. At the same time, reduced epithelial renewal and impaired mucus barrier integrity, which have been reported in older populations, may contribute to reduced stability of commensal microbial communities [70,71]. Zhong et al. (2020) reported that *Blautia* and *Bacteroides* were among the key genera distinguishing metabolically healthy and unhealthy older adults in a cohort of 382 community-dwelling individuals, providing independent support for the role of microbiota composition in healthy versus pathological aging [72].

The age-gradient analysis in the longevity- and age-associated gut microbiome datasets provided additional descriptive evidence of age-related taxonomic remodeling. *Prevotella/Segatella* decreased from 14.99% in the 20–40-year group to 4.34% in the 80–100-year group, while *Faecalibacterium* also showed an age-associated decline. These observations involve taxa previously associated with fiber utilization or SCFA metabolism but do not directly demonstrate reduced fiber fermentation or SCFA production [64]. PICRUSt2 additionally predicted age-associated differences in pathway profiles related to complex carbohydrate degradation and butyrate biosynthesis, with the most pronounced reduction observed in the oldest age group [73]. However, these results represent predicted genomic functional potential inferred from 16S-derived ASV profiles rather than measured pathway expression, metabolic flux, or metabolite concentrations. Because dietary fiber intake, intestinal transit time, and fecal or circulating SCFA concentrations were not measured, the functional implications of these age-associated patterns remain uncertain.

The present age-stratified analyses identified several overlapping genera meeting the predefined abundance criterion in both predefined age strata. However, the present cross-sectional design cannot determine whether these patterns represent within-individual aging-related changes. In particular, medication use, diet, lifestyle, comorbidities, disease severity, geography, and other potential confounders were not consistently available across cohorts. In addition, differences in cohort composition, laboratory procedures, sequencing characteristics, and taxonomic annotation may have contributed to the observed patterns.

The exclusion of *Dorea* from the shared set in the 60–80-year MI group was due to its mean relative abundance falling slightly below the predefined 0.1% descriptive threshold and should therefore be interpreted as a threshold-dependent observation rather than a biological absence. Likewise, age-stratified variation across longevity- and age-associated gut microbiome datasets provides contextual support for age-related microbial changes but does not establish a definitive biological transition between 60 and 80 years [31]. Overall, the present findings should be interpreted as exploratory descriptive observations suggesting that some genus-level microbial patterns may differ across predefined age strata. Confirmation would require longitudinal, multi-cohort studies with harmonized covariate information and direct functional measurements.

**Figure 7:**
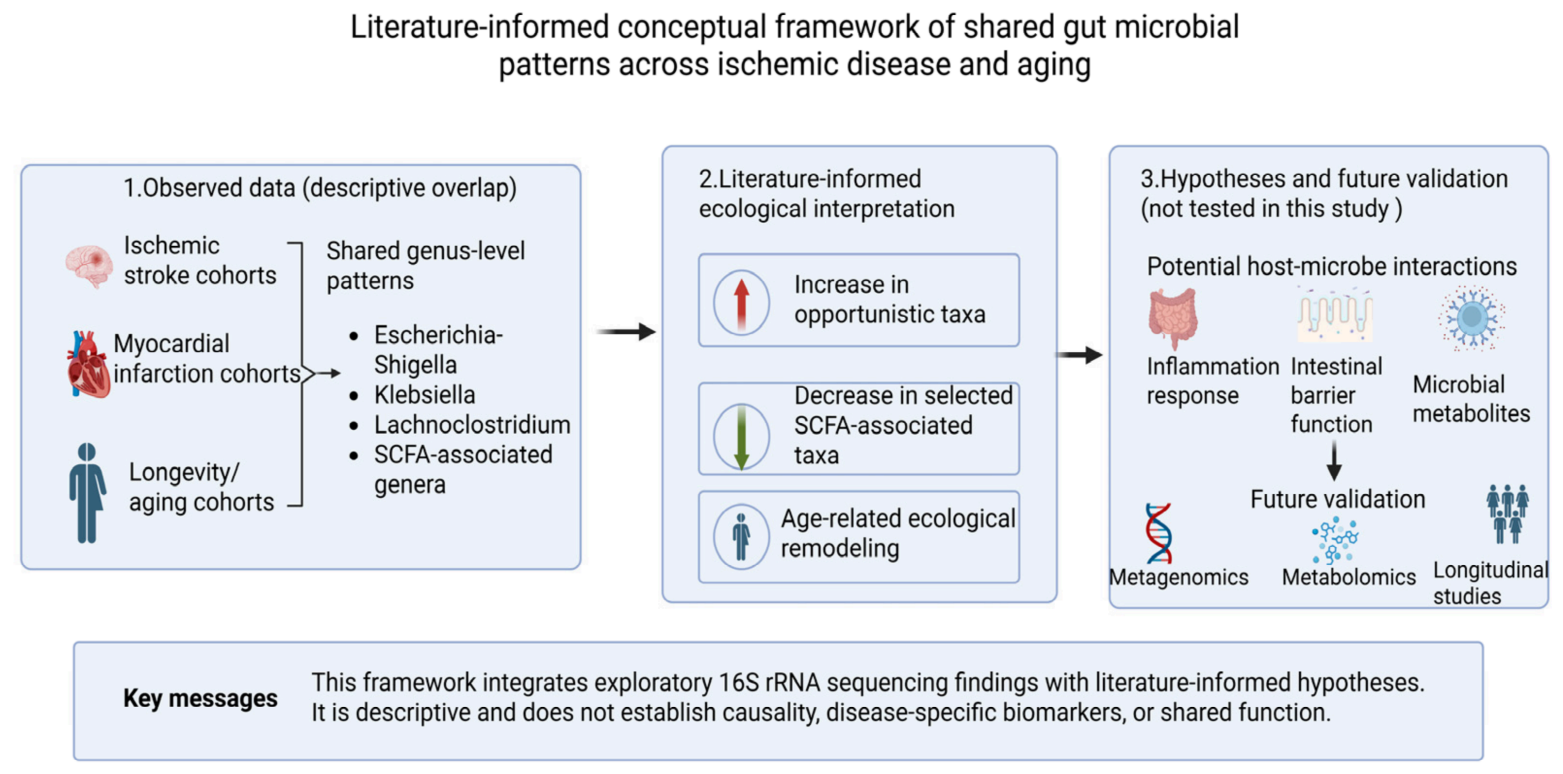
Literature-informed model of age-associated gut microbial remodeling across ischemic disease and longevity-related populations. This model summarizes exploratory microbial patterns across ischemic stroke, myocardial infarction, and longevity-associated populations, including changes in opportunistic-associated taxa and SCFA-associated genera. The proposed relationships are hypothesis-generating and require future validation.

Accordingly, Figure 7 has been reframed as a literature-informed conceptual model rather than a mechanistically validated model. The relationships shown among aging, inflammation, intestinal barrier alterations, SCFA-associated genera, and opportunistic pathogens were not directly tested in the present study. They are presented only as hypotheses that may guide future longitudinal and multi-omics investigations. Supporting age-stratified compositional and predicted functional results are shown (Figure S7; Tables S8, S10 and S11).

### 3.5. Regulatory Properties of Repeatedly Detected Bacterial Genera and Implications for Intervention

The repeatedly detected genera showed reproducible but context-dependent patterns across disease and age-stratified datasets (Figure S8). *Lachnoclostridium* was the only genus consistently retained across all analytical cohorts and age strata, showing a consistent abundance gradient across the analyzed populations, with the highest abundance in MI, followed by stroke and longevity- or age-associated gut microbiome populations (Figure S9). Previous studies have reported divergent responses of *Lachnoclostridium* to dietary or bioactive interventions, including restoration after inulin supplementation in a diabetic model [74], suppression of overgrowth in an obesity model [75], and reduction after capsaicin treatment [76]. These findings suggest that the ecological role of *Lachnoclostridium* is likely context-dependent and may differ across species or strains. Future studies should clarify species- and strain-level functional heterogeneity within this genus, particularly to distinguish potentially beneficial members from taxa associated with pro-inflammatory activity.

*Bacteroides* showed an inverse abundance pattern compared with *Lachnoclostridium*, with higher relative abundance in longevity- and age-associated gut microbiome populations than in stroke and MI cohorts. Enterotoxigenic *Bacteroides* fragilis has been reported to persist under inflammation-associated oxygen-enriched conditions [77], while animal-based diets can promote *Bacteroides* enrichment in humans [78]. Therefore, the observed enrichment of *Bacteroides* in age-associated datasets may reflect both inflammatory ecological restructuring and dietary influences, rather than a uniformly beneficial or detrimental effect.

Age-stratified analyses further showed opposing trajectories for *Escherichia-Shigella* and *Prevotella/Segatella. Escherichia-Shigella* increased progressively with advancing age, whereas *Prevotella/Segatella* declined. Prior studies have shown that capsaicin can promote the clearance of intracellular *Shigella* [79], whereas *Prevotella* strains associated with long-term high-fiber diets are enriched in genes involved in complex carbohydrate degradation [80]. Together, these observations are consistent with the hypothesis that limiting the expansion of opportunistic taxa while preserving fiber-utilizing microbial niches may contribute to maintaining gut ecological stability during aging.

Overall, these genera do not define direct therapeutic targets but instead describe an exploratory pattern of ecological remodeling across disease- and age-associated microbiome contexts. The observed compositional changes involved fiber-utilizing and SCFA-associated taxa together with predicted pathway differences related to carbohydrate fermentation and environmental adaptation [81]. Because these functional predictions do not demonstrate actual SCFA production or metabolic activity, they should be interpreted cautiously. Accordingly, the 60–80-year age group was selected as the primary analytical stratum because ecological differences appeared more evident within this age range, providing a common context for exploratory cross-cohort comparisons across ischemic stroke, myocardial infarction, and age-associated datasets [17].

### 3.6. Limitations

This study has several limitations. First, participant-level metadata were incompletely available in several public datasets. Therefore, several important confounding factors, including medication use, comorbidities, dietary habits, body mass index, geographic origin, and laboratory procedures, could not be fully controlled. In addition, because the external cohorts were identified through targeted searches of public repositories rather than a prospectively planned systematic review, dataset-selection bias cannot be completely excluded. Thus, the present findings should be interpreted as exploratory and hypothesis-generating comparisons across selected IS, MI, and age-associated gut microbiome datasets.

Second, the limited sample size of the internal IS cohort may have reduced the statistical power to detect microbial overlap between ischemic stroke and myocardial infarction, particularly for rare or low-abundance taxa. Accordingly, the shared microbiome framework identified here should be regarded as a preliminary ecological model that requires validation in larger independent cohorts [66].

Third, the cross-sectional design does not allow temporal or causal inference between microbiota alterations and disease progression. Although cohort comparability was improved through age-stratified analyses and a standardized bioinformatics workflow, residual technical heterogeneity may remain because of differences in sample collection procedures, sequencing protocols, geographic or dietary backgrounds, and other study-specific factors. In particular, differences in amplified 16S rRNA variable regions and dataset-specific bioinformatic processing may have influenced taxonomic profiles despite standardized downstream analyses.

Finally, 16S rRNA V3–V4 sequencing provides limited species- and strain-level resolution and cannot fully resolve functional heterogeneity within genera. PICRUSt2-based functional prediction is also inferential and cannot determine gene expression, pathway activity, metabolic flux, or metabolite concentrations [82,83]. Therefore, all SCFA-related interpretations should be considered exploratory. Future multicenter longitudinal studies incorporating shotgun metagenomics, metatranscriptomics, targeted metabolomics, detailed dietary assessment, and independent validation cohorts will be required to determine the biological significance and generalizability of the observed cross-disease microbiome patterns.

## 4. Materials and Methods

### 4.1. Research Design and Statistical Analysis

This exploratory study included one internally recruited ischemic stroke (IS) cohort together with publicly available external IS, myocardial infarction (MI), and age-stratified population datasets. The analytical framework consisted of within-cohort comparisons in the internal IS cohort, independent characterization of each public dataset, and descriptive genus-level comparisons across predefined age strata. Each dataset was processed and analyzed independently according to its amplified 16S rRNA region, sequencing platform, read configuration, quality profile, and available metadata [84]. Feature tables from different datasets, particularly those targeting different or non-overlapping amplicon regions, were not merged [85]. Cross-dataset comparisons were restricted to genus-level relative-abundance profiles generated separately within each dataset.

Baseline IS patients and healthy controls were compared using the Wilcoxon rank-sum test. Treatment-related changes were evaluated only in the 19 patients with matched baseline and follow-up samples using the Wilcoxon signed-rank test. Comparisons involving more than two independent groups were performed using the Kruskal–Wallis test, followed, where applicable, by Dunn’s test. *p* values from multiple taxonomic comparisons were adjusted using the Benjamini–Hochberg false-discovery-rate procedure. Bray–Curtis dissimilarities were evaluated separately within each dataset and visualized using principal coordinate analysis. Differences in community composition were assessed using PERMANOVA with 999 permutations. For paired baseline–follow-up analyses, permutations were constrained by participant ID. Multivariate dispersion was assessed using betadisper and permutation testing before interpreting PERMANOVA results. Analyses were conducted in R version 4.3.

For the descriptive cross-cohort analysis, genera with a mean relative abundance > 0.1% within the corresponding dataset were retained solely as a filtering approach to minimize the influence of extremely low-abundance taxa during qualitative comparisons. This threshold was not used as a statistical significance criterion or as a definition of microbial biomarkers.Overlap among the resulting genus lists was interpreted descriptively as candidate taxa commonly detected across datasets and was not considered evidence of shared disease-associated effects or causal relationships [86]. Because participant-level covariates were not consistently available across public datasets, cross-cohort analyses were descriptive and were not used to estimate covariate-adjusted effects.

### 4.2. Study Cohorts and Public Datasets

The internal IS cohort comprised 54 unique participants, including 33 patients with acute IS and 21 age-matched healthy controls. All patients provided a baseline stool sample before treatment initiation. Among them, 19 provided an additional follow-up sample after approximately 21 days of guideline-based conventional treatment combined with Buyang Huanwu Decoction. Baseline and follow-up samples were linked using deidentified participant identifiers. Thus, the internal cohort comprised 73 stool samples: 33 baseline IS samples, 19 paired post-treatment samples, and 21 healthy-control samples. The post-treatment samples represented repeated observations from the same participants rather than an independent treatment group. Fourteen patients did not provide an evaluable follow-up sample, and missing follow-up values were not imputed. Detailed eligibility criteria, treatment information, sampling procedures, and cohort-level summaries are described and summarized (Table S1).

Public datasets were identified through targeted, non-systematic searches of the NCBI BioProject and Sequence Read Archive databases. The external IS dataset contained 347 samples from participants aged 40–80 years, including 202 samples from participants aged 40–60 years and 145 from those aged 60–80 years. The MI dataset contained 92 samples from participants aged 40–80 years. The longevity- and age-stratified component comprised 2650 samples from multiple public source datasets. Participants were categorized into four age strata: 20–40, 40–60, 60–80, and 80–100 years. The 60–80-year stratum was selected for the primary age-matched descriptive comparison, while the remaining strata were used to characterize age-related microbial patterns. These datasets were population-based longevity or aging studies and did not represent inflammatory bowel disease cohorts.

The search strategy, search date, eligibility criteria, and dataset-selection process are described in the Materials and Methods section. Repository accessions, source publications, disease or population definitions, countries, sequencing platforms, amplified regions, and available metadata are summarized (Table S1). BioProject-BioSample-SRA run-original sample ID mappings and inclusion status are summarized (Table S2).

### 4.3. 16S rRNA Sequencing and Bioinformatics Analysis

The following wet-laboratory procedures apply exclusively to the internal IS cohort. After collection, stool samples were stored at −80 °C until DNA extraction. Microbial DNA was extracted using the QIAamp DNA Stool Mini Kit (QIAGEN, Hilden, Germany) according to the manufacturer’s instructions. The V3–V4 region of the bacterial 16S rRNA gene was amplified using primers 341F (5′-CCTACGGGNGGCWGCAG-3′) and 806R (5′-GGACTACHVGGGTATCTAAT-3′). Triplicate PCRs were performed in 50 μL reactions containing 5 μL of 10× KOD buffer, 5 μL of 2 mM dNTPs, 3 μL of 25 mM MgSO_4_, 1.5 μL of each primer (10 μM), 1 μL of KOD polymerase (TOYOBO Co., Ltd., Osaka, Japan), and 100 ng of template DNA. Cycling conditions were 94 °C for 2 min; 30 cycles of 98 °C for 10 s, 62 °C for 30 s, and 68 °C for 30 s; followed by 68 °C for 5 min. Amplicons were purified, quantified, pooled at equimolar concentrations, and sequenced on an Illumina platform ((Illumina, Inc., San Diego, CA, USA) using paired-end sequencing.

Bioinformatic processing was organized into three streams: the internal IS cohort; the external IS and MI datasets and the longevity- and age-stratified datasets. Raw reads were processed separately within each stream using parameters appropriate for the amplified region, sequencing platform, read configuration, and quality profile. The software versions, exact commands, quality-filtering parameters, feature-generation methods, chimera-removal procedures, taxonomic classifiers, and sample-exclusion criteria are summarized (Table S3).

Feature tables were generated independently within each processing stream and were not merged across datasets targeting different or non-overlapping 16S rRNA regions [84]. Taxonomic annotation was performed separately using the SILVA reference database and the stream-specific classification procedures (Table S3). Non-target sequences were removed according to the corresponding processing protocol. Features lacking reliable genus-level assignments were retained for overall community analyses but excluded from comparisons of named genera. Cross-dataset comparisons were restricted to independently calculated genus-level relative-abundance profiles.

Within each processing stream, sequencing-depth normalization and alpha-diversity analyses were performed separately. The normalization method, minimum read threshold, retained sequencing depth, and excluded samples for each dataset are summarized (Table S4). Genus-level relative abundance was calculated as the proportion of reads assigned to each genus relative to the total retained bacterial reads within the corresponding sample. Alpha-diversity values were not directly compared across independently processed datasets. PICRUSt2 version 2.5 was applied independently to eligible datasets containing at least 30 samples after quality control. Prediction quality was evaluated using the Nearest Sequenced Taxon Index. Predicted KEGG Ortholog, Enzyme Commission, and MetaCyc pathway abundances were analyzed as within-sample relative abundances [87]. Age-associated predicted functional pathway summaries are shown (Table S11). These results were interpreted as exploratory functional predictions rather than direct measurements of microbial function and were not used to infer biological mechanisms or causal relationships.

## 5. Conclusions

This work should be interpreted as an exploratory and hypothesis-generating descriptive study, not as a validation study of disease-specific microbial signatures or mechanisms. Using a predefined hierarchical comparison framework and standardized downstream analyses, we identified candidate genus-level overlap across ischemic stroke (IS), myocardial infarction (MI), and age-stratified or longevity-associated gut microbiome datasets. At the cross-cohort level, 15 genera were detected in both internal and external stroke cohorts within the 60–80-year stratum. At the cross-disease level, seven genera met the predefined abundance criterion in both external IS and MI datasets in the 40–60-year stratum, and six genera were retained across the stroke and MI datasets in the 60–80-year stratum. These overlaps provide a descriptive framework for future validation rather than evidence of a conserved disease mechanism.

Within this descriptive framework, *Lachnoclostridium* was retained across analytical levels and showed a cross-dataset abundance gradient. *Escherichia-Shigella* and *Klebsiella* were repeatedly detected across disease and age-stratified datasets. Selected short-chain-fatty-acid-associated genera, including *Blautia* and *Roseburia*, showed lower relative abundance in some comparisons but were not uniformly depleted across all datasets. Although *Faecalibacterium* was not retained in the final intersection set, its lower relative abundance was observed in selected IS comparisons [88]. These findings describe compositional patterns only and should not be interpreted as evidence of shared microbial function, altered short-chain fatty acid biosynthesis, treatment efficacy, or causal disease mechanisms.

In the limited paired follow-up analysis, *Escherichia-Shigella* decreased in relative abundance, whereas *Faecalibacterium*, *Lachnoclostridium*, and *Blautia* showed variable changes [89]. Because the internal cohort was small and no direct functional measurements were obtained, these observations remain hypothesis-generating. Any mechanistic models discussed in this manuscript are literature-informed hypotheses derived from exploratory data; they were not directly tested and require future validation. Larger, well-characterized multicenter cohorts with standardized protocols, longitudinal sampling, shotgun metagenomics, metatranscriptomics, metabolomics, and experimental studies will be needed to determine whether the candidate genera represent biologically meaningful cross-disease microbial patterns.

## Data Availability

The raw 16S rRNA gene amplicon sequencing data generated from the internal ischemic stroke cohort have been deposited in the National Omics Data Encyclopedia (NODE) database (https://www.biosino.org/node/sample/detail/OES00413247, accessed on 30 July 2026) under accession number OES00413247. The deposited sequencing data have been de-identified and do not contain personally identifiable information. All procedures involving human participants were conducted in accordance with institutional ethical approval and the original informed-consent framework. The external ischemic stroke, myocardial infarction, and longevity- and age-associated gut microbiome datasets analyzed in this study were obtained from previously published studies and publicly available repositories. Accession numbers, source publications, cohort information, bioinformatic processing summaries, and processed genus-level abundance matrices are provided in the Materials and Methods and Supplementary Tables S1–S8. Community-structure statistics, age-associated taxonomic analyses, predicted functional results, and supporting visualizations are provided in Supplementary Tables S9–S11 and Supplementary Figures S1–S9. Additional processed data and analysis materials not included in the public repository may be available from the corresponding author upon reasonable request, subject to ethical and institutional requirements.

## Supplementary Materials

The following supporting information can be downloaded at: https://www.mdpi.com/article/10.3390/ijms27157020/s1.

## Abbreviations

The following abbreviations are used in this manuscript:

MI	Myocardial Infarction
IS	Ischemic Stroke
PCoA	Principal Coordinates Analysis
LPS	Lipopolysaccharide
SCFA	Short-Chain Fatty Acid
PERMANOVA	Permutational Multivariate Analysis of Variance
ASV	Amplicon Sequence Variant
