# Supplementary material for: Convergent Gut Microbiome Remodeling Across Ischemic Stroke, Myocardial Infarction, and Longevity Reveals a Shared Ecological Signature of Aging and Disease
Source: Int J Mol Sci. 2026 Aug 5;27(15):7020. doi: 10.3390/ijms27157020 (PMC13467180; doi:10.3390/ijms27157020)
Supplement: Supplementary file 1 [file ijms-27-07020-s001.zip › Figure S1. Alpha diversity of the external ischemic stroke cohort.pdf]

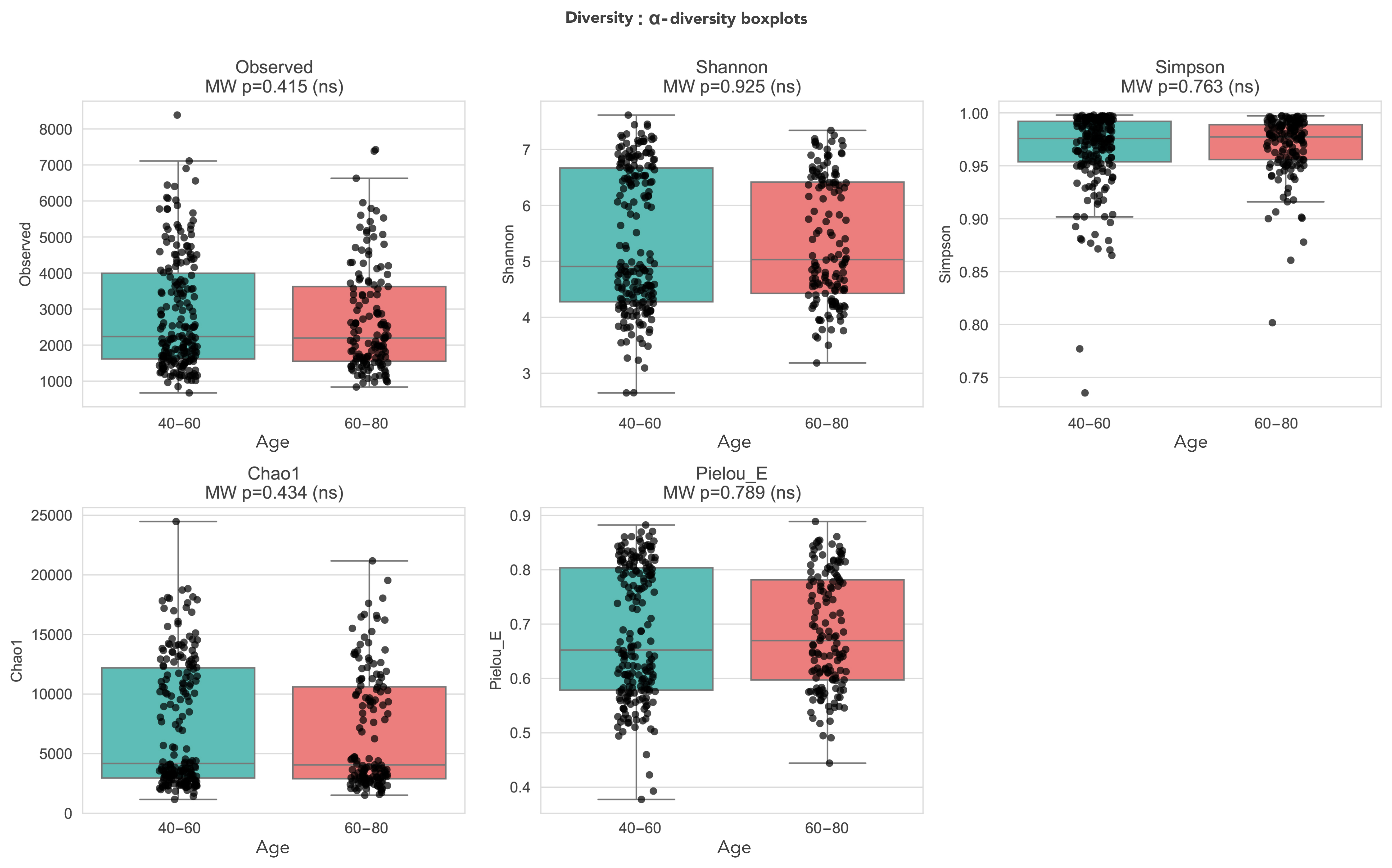

Diversity : α-diversity boxplots
Observed
MW p=0.415 (ns)
Shannon
MW p=0.925 (ns)
Simpson
MW p=0.763 (ns)
Chao1
MW p=0.434 (ns)
Pielou_E
MW p=0.789 (ns)
Age
40–60
60–80
