# Supplementary material for: Convergent Gut Microbiome Remodeling Across Ischemic Stroke, Myocardial Infarction, and Longevity Reveals a Shared Ecological Signature of Aging and Disease
Source: Int J Mol Sci. 2026 Aug 5;27(15):7020. doi: 10.3390/ijms27157020 (PMC13467180; doi:10.3390/ijms27157020)

Diversity : α-diversity boxplots

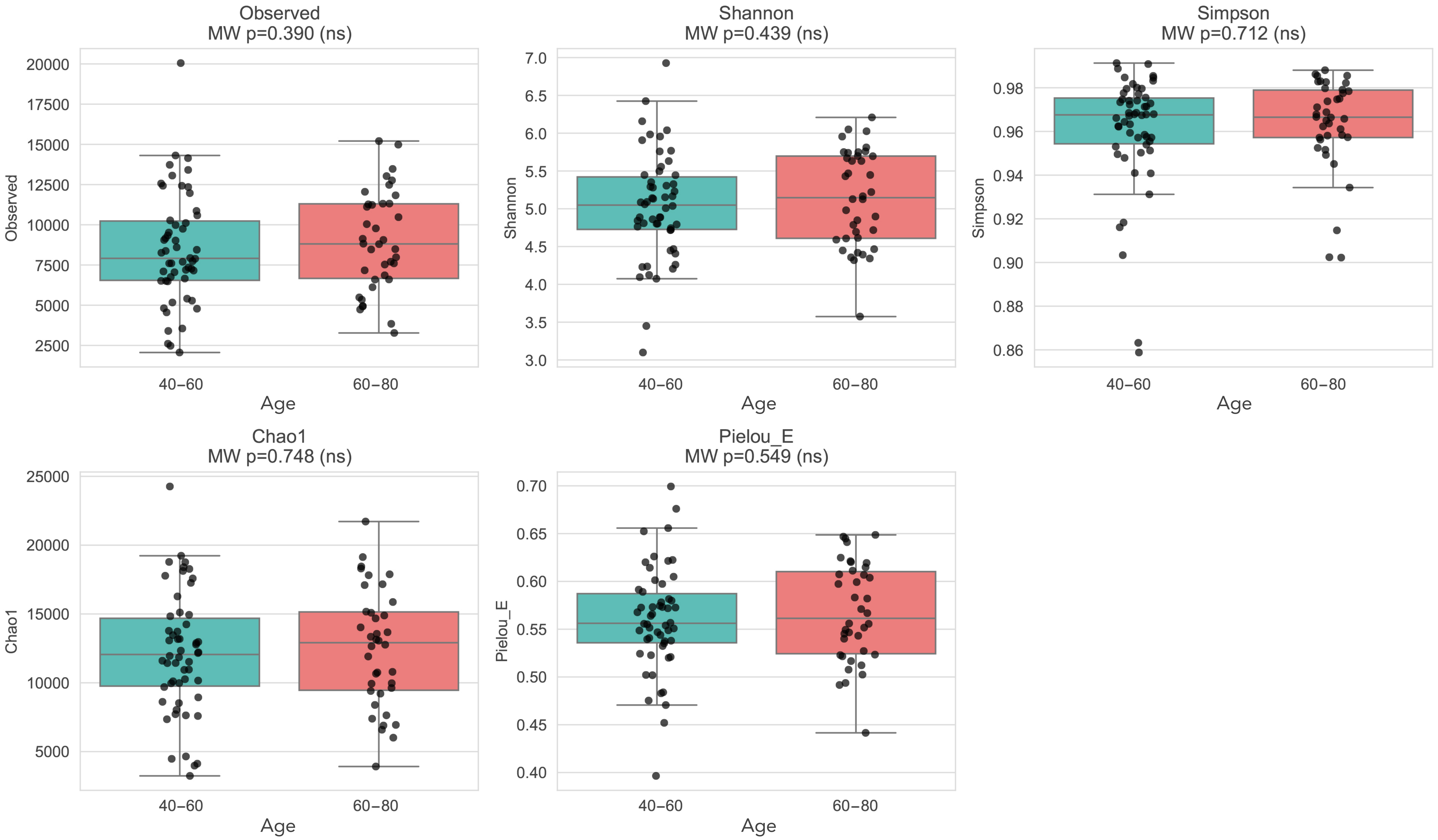

Supplement: Supplementary file 1 [file ijms-27-07020-s001.zip › Figure S2. Alpha diversity of the MI cohort.pdf]
