# Supplementary material for: Convergent Gut Microbiome Remodeling Across Ischemic Stroke, Myocardial Infarction, and Longevity Reveals a Shared Ecological Signature of Aging and Disease
Source: Int J Mol Sci. 2026 Aug 5;27(15):7020. doi: 10.3390/ijms27157020 (PMC13467180; doi:10.3390/ijms27157020)
Supplement: Supplementary file 1 [file ijms-27-07020-s001.zip › Figure S3. Principal coordinate analysis of the external ischemic stroke cohort.pdf]

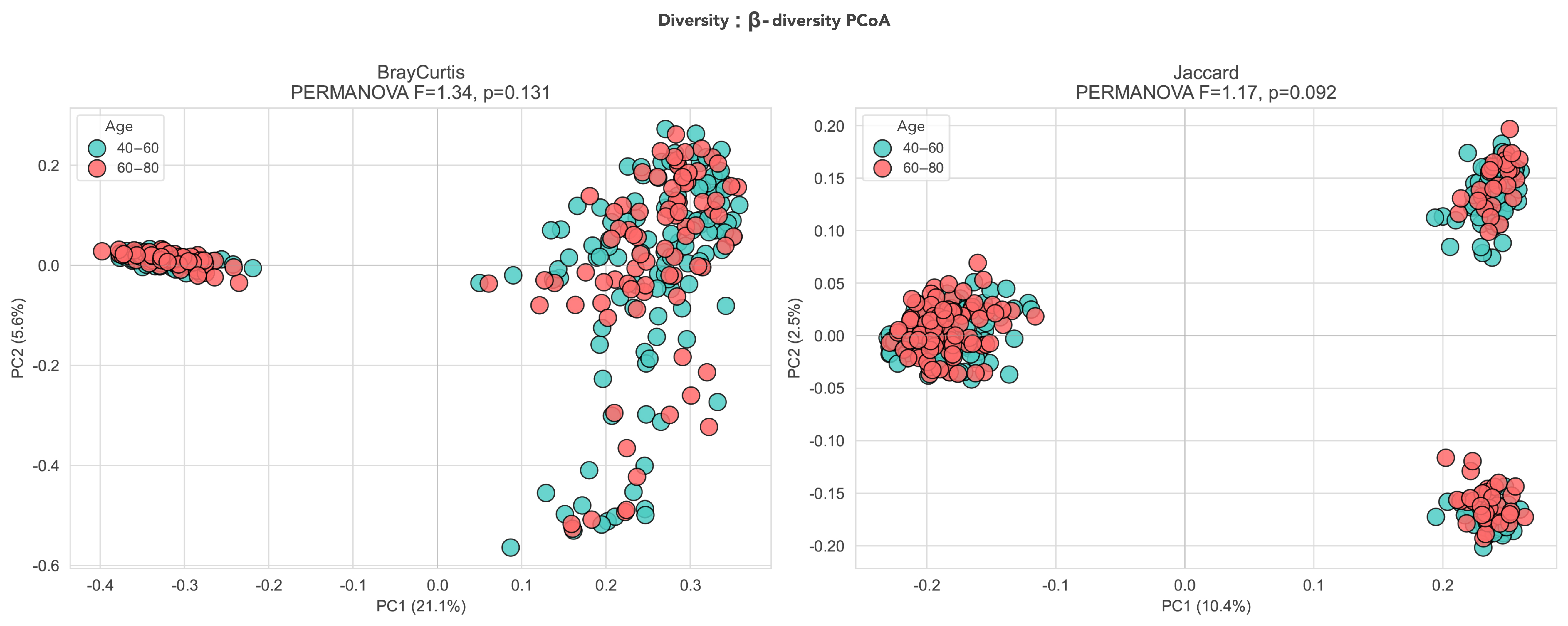
Diversity : β- diversity PCoA
BrayCurtis
PERMANOVA F=1.34, p=0.131
Age
40–60
60–80
PC2 (5.6%)
PC1 (21.1%)
0.2
0.0
-0.2
-0.4
-0.6
-0.4
-0.3
-0.2
-0.1
0.0
0.1
0.2
0.3
Jaccard
PERMANOVA F=1.17, p=0.092
Age
40–60
60–80
PC2 (2.5%)
PC1 (10.4%)
0.20
0.15
0.10
0.05
0.00
-0.05
-0.10
-0.15
-0.20
-0.2
-0.1
0.0
0.1
0.2
