# Supplementary material for: Convergent Gut Microbiome Remodeling Across Ischemic Stroke, Myocardial Infarction, and Longevity Reveals a Shared Ecological Signature of Aging and Disease
Source: Int J Mol Sci. 2026 Aug 5;27(15):7020. doi: 10.3390/ijms27157020 (PMC13467180; doi:10.3390/ijms27157020)
Supplement: Supplementary file 1 [file ijms-27-07020-s001.zip › Figure S4. Principal coordinate analysis of the MI cohort.pdf]

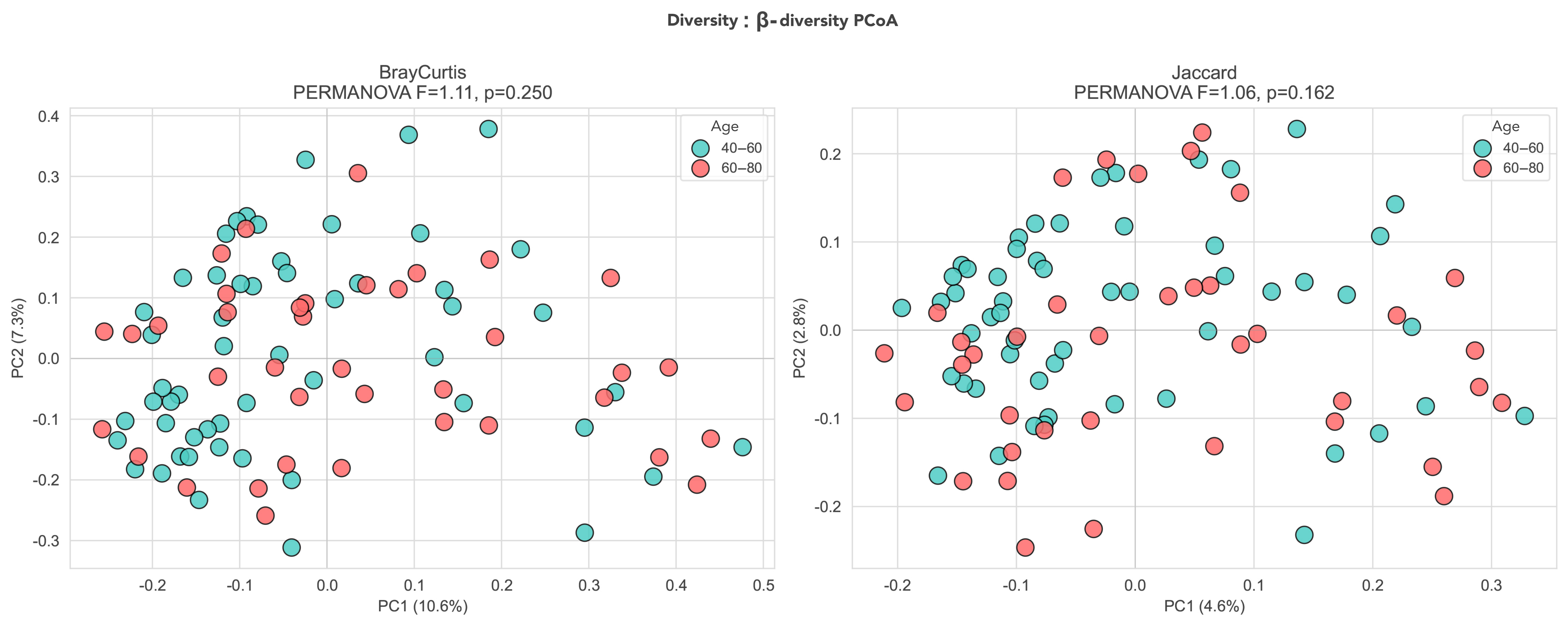
Diversity : β-diversity PCoA
BrayCurtis
PERMANOVA F=1.11, p=0.250
Age
40–60
60–80
PC2 (7.3%)
PC1 (10.6%)
0.4
0.3
0.2
0.1
0.0
-0.1
-0.2
-0.3
-0.2
-0.1
0.0
0.1
0.2
0.3
0.4
0.5
Jaccard
PERMANOVA F=1.06, p=0.162
Age
40–60
60–80
PC2 (2.8%)
PC1 (4.6%)
0.2
0.1
0.0
-0.1
-0.2
-0.2
-0.1
0.0
0.1
0.2
0.3
