# Supplementary material for: Convergent Gut Microbiome Remodeling Across Ischemic Stroke, Myocardial Infarction, and Longevity Reveals a Shared Ecological Signature of Aging and Disease
Source: Int J Mol Sci. 2026 Aug 5;27(15):7020. doi: 10.3390/ijms27157020 (PMC13467180; doi:10.3390/ijms27157020)
Supplement: Supplementary file 1 [file ijms-27-07020-s001.zip › Figure S7. Genus-level microbial composition across longevity and age-stratified gut microbiome datasets.pdf]

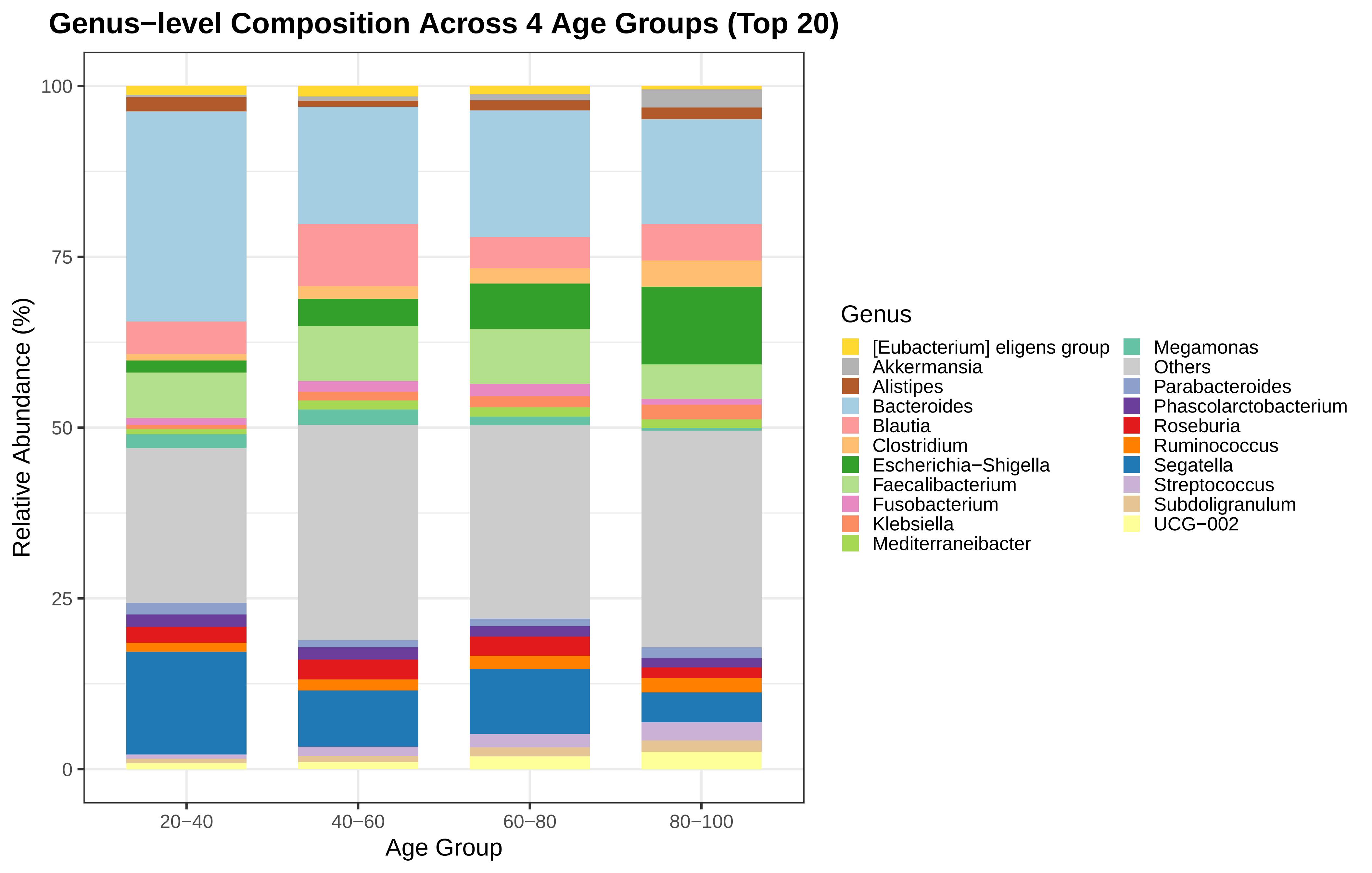
Genus−level Composition Across 4 Age Groups (Top 20)
Relative Abundance (%)
100
75
50
25
0
20−40
40−60
60−80
80−100
Age Group
Genus
[Eubacterium] eligens group
Akkermansia
Alistipes
Bacteroides
Blautia
Clostridium
Escherichia−Shigella
Faecalibacterium
Fusobacterium
Klebsiella
Mediterraneibacter
Megamonas
Others
Parabacteroides
Phascolarctobacterium
Roseburia
Ruminococcus
Segatella
Streptococcus
Subdoligranulum
UCG−002
