# Supplementary material for: Convergent Gut Microbiome Remodeling Across Ischemic Stroke, Myocardial Infarction, and Longevity Reveals a Shared Ecological Signature of Aging and Disease
Source: Int J Mol Sci. 2026 Aug 5;27(15):7020. doi: 10.3390/ijms27157020 (PMC13467180; doi:10.3390/ijms27157020)
Supplement: Supplementary file 1 [file ijms-27-07020-s001.zip › Figure S8. Heatmap of retained overlapping genera across disease and age-stratified gut microbiome datasets.pdf]

Retained overlapping genera across disease and age-stratified gut microbiome datasets

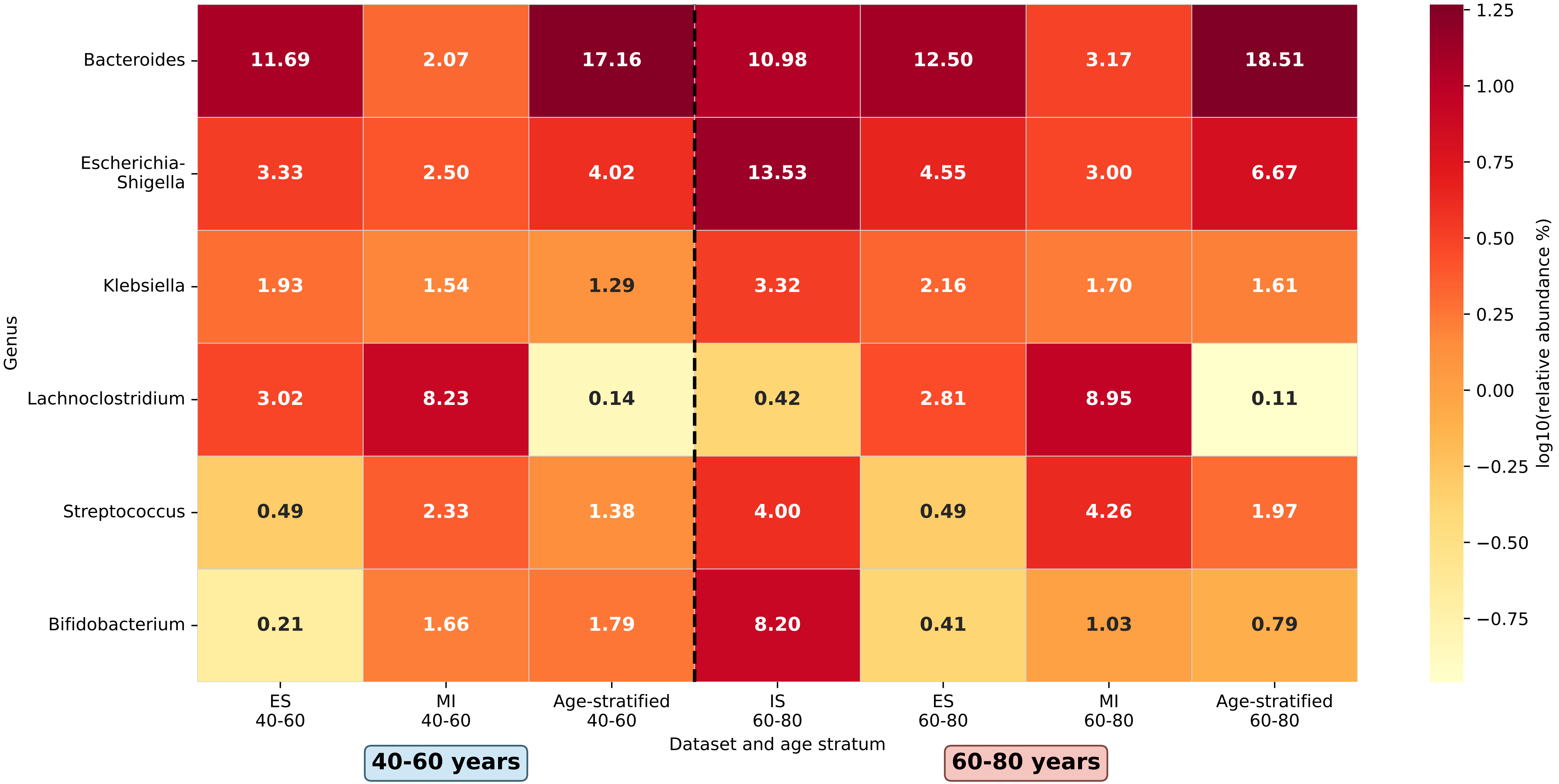

IS, internal ischemic stroke; ES, external ischemic stroke; MI, ACS/MI cohort. Values are mean relative abundance (%).
Overlap reflects the predefined abundance filter and is descriptive, not a formal prevalence-defined community.
