# Supplementary material for: Convergent Gut Microbiome Remodeling Across Ischemic Stroke, Myocardial Infarction, and Longevity Reveals a Shared Ecological Signature of Aging and Disease
Source: Int J Mol Sci. 2026 Aug 5;27(15):7020. doi: 10.3390/ijms27157020 (PMC13467180; doi:10.3390/ijms27157020)
Supplement: Supplementary file 1 [file ijms-27-07020-s001.zip › Figure S9. Relative abundance gradient of Lachnoclostridium across analyzed datasets.pdf]

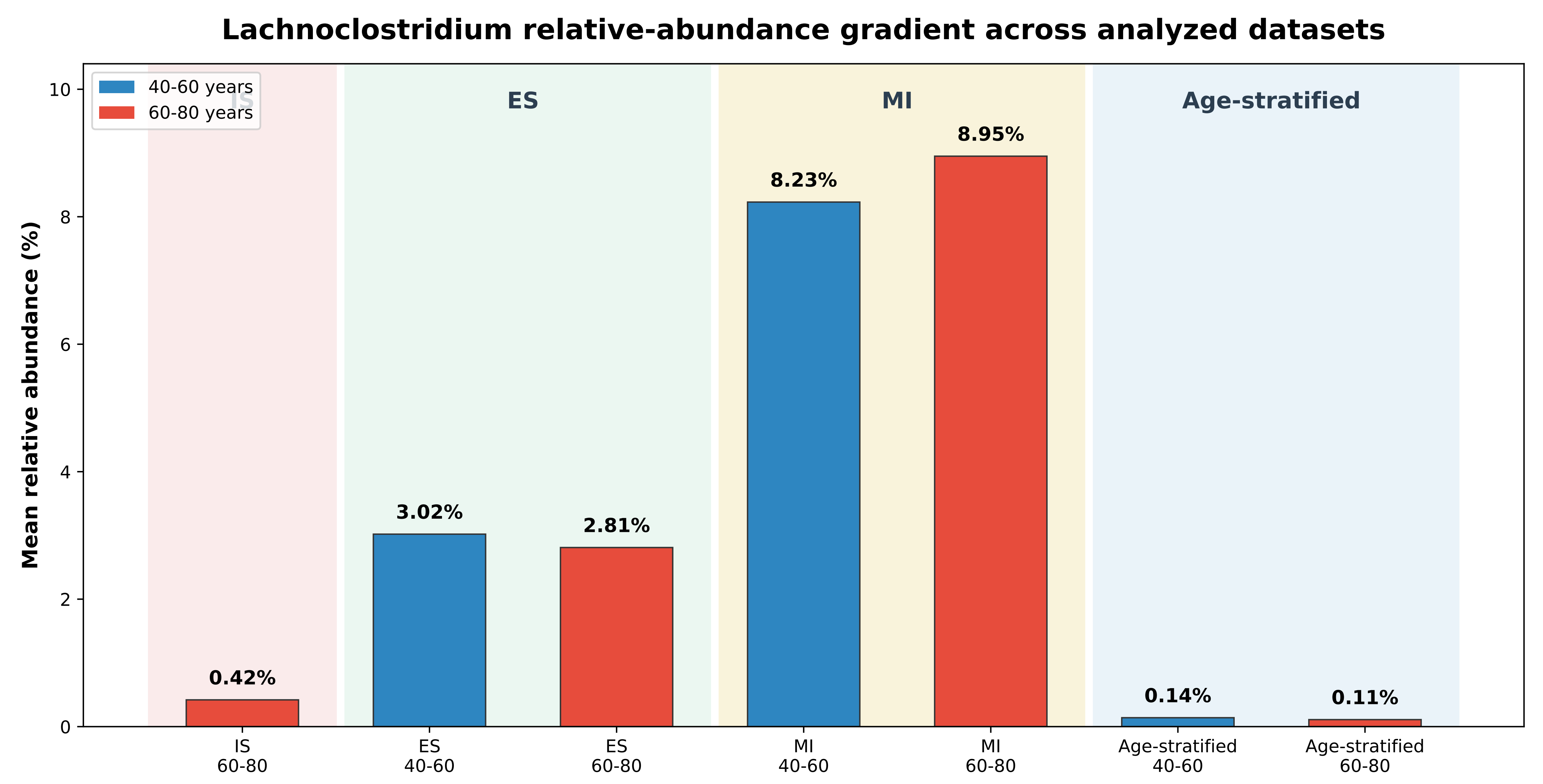

The gradient is a descriptive abundance pattern among retained genera and should not be interpreted as a causal or disease-specific biomarker signal.
